# Potato Berries as a Valuable Source of Compounds Potentially Applicable in Crop Protection and Pharmaceutical Sectors: A Review

**DOI:** 10.1021/acs.jafc.4c03071

**Published:** 2024-07-06

**Authors:** Marília Bueno da Silva, Anika Wiese-Klinkenberg, Björn Usadel, Franziska Genzel

**Affiliations:** † Institute of Bio- and Geosciences (IBG-4: Bioinformatics), Bioeconomy Science Center (BioSC), CEPLAS, Forschungszentrum Jülich GmbH, 52425 Jülich, Germany; ‡ Faculty of Mathematics and Natural Sciences, CEPLAS, Institute for Biological Data Science, Heinrich Heine University Düsseldorf, 40225 Düsseldorf, Germany

**Keywords:** potato berries, glycoalkaloids, α-solanine, α-chaconine, bioeconomy

## Abstract

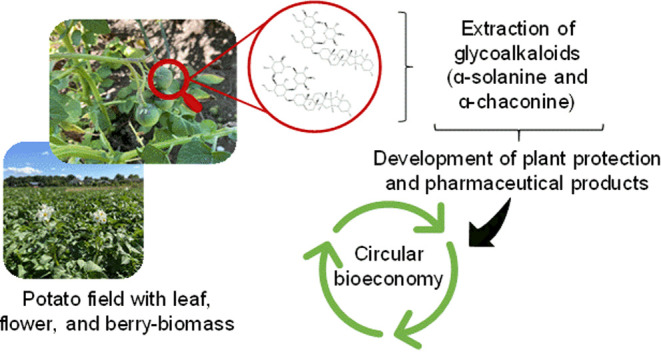

Potato (*Solanum tuberosum*) is a major agricultural crop cultivated worldwide. To meet market demand, breeding programs focus on enhancing important agricultural traits such as disease resistance and improvement of tuber palatability. However, while potato tubers get a lot of attention from research, potato berries are mostly overlooked due to their level of toxicity and lack of usefulness for the food production sector. Generally, they remain unused in the production fields after harvesting the tuber. These berries are toxic due to high levels of glycoalkaloids, which might confer some interesting bioactivities. Berries of various solanaceous species contain bioactive secondary metabolites, suggesting that potato berries might contain similarly valuable metabolites. Therefore, possible applications of potato berries, e.g., in the protection of plants against pests and pathogens, as well as the medical exploitation of their anti-inflammatory, anticarcinogenic, and antifungal properties, are plausible. The presence of valuable compounds in potato berries could also contribute to the bioeconomy by providing a novel use for otherwise discarded agricultural side streams. Here we review the potential use of these berries for the extraction of compounds that can be exploited to produce pharmaceuticals and plant protection products.

## 1 Introduction

Food production must increase to feed a population that is projected to reach 9.7 billion people by 2050, but this must be achieved without damaging the environment.^1^ Because traditional agricultural practices are known for lacking the sustainability factor due to the intensive use of mechanical implements and dependence on synthetic pest control products, it is therefore important to find more sustainable options for agricultural production and management of agricultural residues,^2^ which is a component of UN Sustainable Development Goal 12.^3^ One way to promote sustainable agriculture is through circular bioeconomy, to which agricultural production can be a pivotal ally. The valorization of byproducts generated pre- and postharvest is responsible for increasing sustainability in primary production,^4^ especially if these byproducts are associated with biotechnology development and improvement.^5^ Raw materials from plants or animals that are not converted into food are considered agricultural wastes, which are mainly divided into crop residues, food processing waste, and livestock waste. Residues from the cultivation of fruits and vegetables contain a high level of nutrients and bioactive compounds, which can be extracted and used in the pharmaceutical and/or nutraceutical industries or to control agricultural pests and diseases.^6^

Among the examples of agriculture as one of the main actors in a circular bioeconomy are the use of almond hulls and shells for the control of the nematode *Pratylenchus vulnus*(7) or walnut husks and shells as a source of antioxidants and herbicides.^8^ In the food industry, Chamorro et al. studied the use of kiwi residues (such as discarded fruits, skin, and seeds) as feedstock for pharmaceutical and nutraceutical additives.^9^ On the same line, dos Santos et al. studied the potential bactericidal effect of garlic peel extracts against *Staphylococcus aureus* and *Listeria monocytogenes*.^10^ Sifola et al. and Myo and Khat-Udomkiri showed different extraction techniques to increase the yield efficiency of polyphenols from tobacco stalks and coffee pulp.^11,12^ Bagasse of blueberry or some Peruvian berries (elderberry, blackberry, goldenberry, and blueberry) have also been studied by Ferreira et al. and Rojas-Ocampo et al. with the purpose of extraction of polyphenols and anthocyanins.^13,14^ Furthermore, it was shown that it is possible to increase the concentration of flavonoids and other secondary metabolites of interest by targeted application of abiotic stress in residual horti- and agricultural plant biomass for further valorization of the horticultural production.^15−18^ Within solanaceous plants, the circular bioeconomy of tomato biomass is widely studied, either to develop new biodegradable composite thermoplastic material^19^ or to extract bioactive compounds from residual plant byproducts.^16,17^

Potato (*Solanum tuberosum*) is one of the major global vegetable crops^20^ being used for fresh market or food processing industry (e.g., chips and starch production).^21^ This solanaceous crop is cultivated in more than 100 countries with a total production of more than 376 million tonnes harvested from more than 18 million hectares in 2021.^22^ The production and commercialization of potatoes has been facilitated by their ease of planting and handling, combined with the development of new varieties that produce larger tubers, with low levels of glycoalkaloids, and are better adapted to regions with long days, thus increasing overall yields.^23,24^ Highest yields are currently achieved in Kuwait, New Zealand, and in the USA (51.92, 50.83, and 49.07 t/ha in 2021, respectively), whereas the lowest are reported by the Central African Republic, Timor-Leste, and Eritrea with a summed yield of 2.63 kg/ha in 2021.^22^ The global average potato consumption is ∼33 kg per capita/year, which is equivalent of 261.1 kJ per capita/day.^22^ Potato is a staple food in many countries and has a positive impact on food security.^25^ Potato farming also helps to address poverty, particularly in areas dominated by subsistence agriculture, by generating jobs and additional income while reducing price volatility.^20,23^

The intense use of potato as raw material is often linked to a large production of waste. For instance, the process of peeling alone is responsible for up to 10% of the potato waste generated.^26^ The starch production, for example, is responsible for the most varied byproducts (potato wastewater, potato pulp, and potato peel) generated throughout the potato supply chain.^27^ Potato wastewater is characterized by the presence of fibers, minerals (potassium, phosphorus), and proteins such as patatins and amino acids.^27^ When deproteinated and enriched by a source of carbon (glycerol, glucose, lactose), potato wastewater can be used as a nitrogen source for production of lipids and carotenoids by yeast.^28,29^ Another important byproduct of potato industrial sector (especially for production of chips) is potato peel. Due to its content in polyphenols and fibers, different applications for this byproduct are described in the literature.^27,30,31^ For example, Haleem et al. studied the applicability of potato peels as flocculant agent in livestock wastewater. Potato peels presented a suspended solid removal efficiency of 72 and 164 mg SS/mg flocculant in swine and dairy wastewater, respectively.^5^ In general, these numbers were greater than the commercial flocculants, which ranged from 0.452 to 187 mg SS/mg flocculant for swine and 0.67–21.1 mg SS/mg flocculant for dairy.^5^ Potato peel has also been discussed and investigated as residual biomass for the extraction of steroidal alkaloids by pressurized liquid extraction.^32^ Samotyja also studied the extract of potato peel and the evaluation of its antioxidant capacity due to the high amount of phenolic compounds in its composition.^33^ Use of potato agricultural residues for extraction of bioactive and industrially relevant metabolites has been so far solely discussed for solanesol in potato leaves, a triterpenoid with bioactivities relevant for pharmaceutical use and additionally used as an industrial precursor for coenzyme Q10 and vitamin K analogues.^34^

While several studies have proposed methods to deal with the wasted pulp and peel,^27,35−37^ little is mentioned about other byproducts. Potato cultivation also generates a large volume of biomass that remains in the fields following the harvest of tubers. This biomass, composed of leaves, stems, flowers, and true fruits (berries), is also full of compounds with interesting biological activities, such as glycoalkaloids and phenolic compounds,^38,39^ but the potential of these byproducts to add value to the agricultural production has been largely overlooked by the scientific community. In this review, we discuss the potential use of berries from potato fields for the extraction of important compounds for the pharmaceutical industry and for the development of plant protection products, improving the sense of circular bioeconomy for this crop.

## 2 Agricultural Side Streams of Potato Tuber Production

### 2.1 General Biology of Potato

Potato plants are divided into two main parts: an aboveground part with stems, foliage, and reproductive organs, and a belowground part composed of roots, stolons, and edible tubers that can vary in shape and color.^40,41^ The life cycle of a potato plant begins with tuber sprouting, followed by plant emergence, with stems developing from mother tubers and forming green aerial foliage with compound leaves. Meanwhile, stolons are formed below ground, and tuberization is induced. In the upper part, the complete development of the main stem is characterized by the presence of a primary terminal inflorescence. Afterward, additional lateral stems develop, forming new inflorescences in parallel with tuber development.^40,42^ Usually, potato inflorescences generate flowers under environmental parameters such as a light regime of 14–18 h (day–night) and a temperature ranging from 15 to 20 °C,^43^ and these flowers differ in color depending on the variety.^44^ The presence of flowers allows two different types of pollination: autogamy and cross pollination by insects or birds. Once the pollination is successful, the true potato fruit is formed, also known as the potato berry.^41,44^ After tuberization, a shift in the assimilation of nutrients results in the cessation of aerial growth and only the tubers continue to accumulate assimilates.^42^

### 2.2 Potato Berries

Potato berries are small “tomato-like” fruits that vary in diameter (0.5–2.0 cm) and weigh approximately an average of 5 g. Berry setting occurs when open flowers carrying fertile gametes are pollinated and the flowers are not subsequently aborted. Usually, berries appear 1.5 months after pollination.^45^ Studies with “true potato seeds” are mostly from the 1990s, and they show that a combination of genetic, morphological, and environmental factors inhibits berry setting in potato populations.^46−49^

Seeds produced in the potato berries were widely used to produce potatoes in the past due to their high level of cleanliness and low cost.^50,51^ However, as the demand for potato increased, breeders shifted to vegetative propagation, adding new agronomic traits and increasing the stability of potato production.^45,52^ This change in strategy of production resulted in a loss of genetic diversity due to factors such as tetraploid potato breeding that increased sterility levels and inbreeding depression and traditionalism of potato consumers that want “more of the same” in terms of cultivar choice and final products preference.^45,53^ Pollen viability, translated as “the ability to produce seeds when used in crosses”,^45^ is a critical criterion for breeding programs. Sterility in potato populations can also occur due either to the absence of pollen production or to a low rate of pollen discharge or to deformities in anthers, which impair pollination and fruit setting.^54^ Low pollen viability is caused by frequent cytoplasmic male sterility, a failure promoted by an interaction between the nuclear and mitochondrial genomes.^52,55^ This type of sterility is often recorded in American and European potato plants from the species *S. tuberosum* due to the presence of the predominant type-T cytoplasm.^54^ Furthermore, high rates of sterility and the lack of seed fruits also influence current breeding programs, hindering the selection and accumulation of interesting new traits that are not transmitted by pollen.^52^ Environmental factors such as temperature and photoperiod also influence flower opening and fruit setting.^45,56^ There is a negative correlation between hours of light exposure and the percentage of berries formed, especially if high temperatures persist after flower opening, which leads to the abortion of flowers.^57,58^

Recently, modern potato breeding has given more attention to the use of diploid potato hybrids, which revives the idea of incrementing berry production.^59^ This happens because hybrid breeding uses true potato seed (from berries) as initial propagation material, presenting easier logistics when compared to traditional potato breeding,^60^ with positive points of facilitated production, transport, and storage, especially important for the potato production in developing countries.^59^

In practice, the use of diploid potatoes decreases the genetic complexity and speeds up the introduction of desirable traits, such as disease resistance and increased suitability for industry processing, and the removal of undesirable traits, as well as reduces the chances of forming generations with accumulated tuber-borne diseases.^61,62^ In addition, hybrids’ performance can be easily predicted by the performance of the parents.^59,63^

Besides the improved breeding speed, advantages of working with diploid potatoes are also the reduced population size required for mapping, easiness to determine expected ratios, and the possibility of working with tools (SNP analysis platforms) developed for diploid species. In terms of genetic diversity, wild and cultivated potatoes are sexually compatible at diploid levels, generating hybrids with good agronomic value.^64^

To address the berry setting problem with diploid potatoes, Bradshaw explains that self-incompatibility can be overcome, either through the use of a self-incompatibility inhibitor gene (Sli) originally found in *Solanum chacoense*, or through knockout mutations in the S-locus.^65^ The first option was successfully tested by Lindhout et al. by crossing diploid potatoes with an accession of *S. chacoense* carrying the Sli gene, self-ing F1 progenies, and originating F2 progenies with high vigor, good agronomic traits, and increased berry production when self-pollinated.^66^

The production of flowers, berries, and seeds has also been investigated in relation to the growth of the vine or tuber yield and indicated a negative influence of flowering and berry production on both, dependent on environment and genotype.^67,68^ This is explained by a competition for photosynthates.^45,67^ It was also hypothesized that the breeding for high yield cultivars counter selected or omitted breeding for highly flowering and fruiting cultivars.^68,69^

The European Cultivated Potato Database (ECPD) contains information on more than 5.654 cultivars from 26 contributors,^70^ of which for 1.257 there is information on the berry frequency of the cultivars ranging from “no berries” to “very frequent”. Table 1 shows a small selection of 21 cultivars currently grown in EU,^77^ USA, Brazil, and Canada.^71−77^

**Table 1:** Description of Different Cultivars Currently Grown in EU,^77^ USA, Brazil, and Canada^71−77^ According to Their Berry Presence Listed in the European Cultivated Potato Database^a^,^70^

variety	approved in	usability	berry presence	maturity	tuber yield potential	french fry suitability	crisp suitability	starch content	origin
Atlantic	1976	industry	occasional to frequent	intermediate to late	high to very high	good to very good	good	low to high	USA
Avenir	2022	fresh market	rare to occasional	early to intermediate	high to very high	unknown	unknown	low to medium	Netherlands
Berber	1983	fresh market	occasional to frequent	very early to intermediate	high to very high	poor to good	poor to good	low	Netherlands
Cara	1972	fresh market	occasional to very frequent	intermediate to very late	high to very high	poor	poor	low	United Kingdom/Ireland
Concordia	2008	fresh market	frequent	intermediate	medium to very high	unknown	unknown	medium to high	Germany
Désirée	1962	fresh market	occasional to very frequent	intermediate to late	medium to very high	moderate to very good	poor to good	low to high	Netherlands
Diamant	1982	fresh market	rare to occasional	intermediate to late	high to very high	unknown	unknown	medium	Netherlands
Granola	1975	fresh market	very frequent	intermediate to late	medium to high	poor to good	poor to moderate	very low to medium	Germany
Hydra	2022	industry	rare to occasional	early to intermediate	high	poor to moderate	poor to moderate	low to medium	Germany
Innovator	1998	industry	rare to occasional	early	high to very high	good	unknown	unknown	Netherlands
Kardal	1988	industry	rare to occasional	late to very late	high to very high	poor	poor to moderate	high to very high	Netherlands
Omega	2004	fresh market	frequent	early to intermediate	medium to high	unknown	unknown	medium	Germany
Quarta	1979	fresh market	occasional	intermediate	medium to high	unknown	unknown	low to medium	Germany
Ranger Russet	1991	fresh market and industry	no berries	late	unknown	good	good	unknown	USA
Russet Burbank	1908	industryfresh market	no berries to rare	very late to intermediate	medium to very high	moderate to very good	moderate	medium to high	USA
Sandra	2019	fresh market	rare	intermediate	medium to high	poor	poor	very low to medium	Germany
Saturna	1970	fresh market	occasional to frequent	intermediate to late	low to high	poor	good to very good	medium to very high	Netherlands
Shepody	1980	industry	no berries	early to intermediate	low to high	poor to very good	moderate to good	medium to high	Canada
Snowden	1990	industry	no berries	intermediate to late	high to very high	unknown	very good	high to very high	USA
Umatilla Russet	1998	industry	no berries	late	unknown	good	unknown	unknown	USA
Victoria	1997	fresh market	occasional	early to intermediate	high to very high	good	unknown	unknown	Netherlands

a Source: Adapted from the ECPD^70^ and Potato Pedigree Database.^78^ “Unknown” means no information in the ECPD.^70^

The tuber production potential of the potato varieties listed in Table 1 seems to be independent of the frequency of berries, and their production does not necessarily imply a lower productivity. According to the table, varieties that have a high frequency of berries are also varieties that have either a high and/or very high productivity. However, previous studies^67,79^ demonstrated a negative effect on tuber yield with more than 20% reduction when plants were allowed to set berries.

Berry productivity shows a tendency to vary between potato varieties directed either to fresh consumption or industrial processing, some of them are described in Table 1. Potato varieties intended for fresh consumption, e.g., Désirée and Granola, are classified with occasional to very frequent berry production (Sandra as one exception with rare berry production), while varieties such as Innovator, which are cultivated to supply the industrial sector with the production of French fries and chips,^80^ tend to present rare to occasional berry production. The cultivars from USA and Canada, however, produce no or rarely berries, with the exception of Atlantic, also grown in Brazil, which has higher berry occurrence. Parameters related to potato usability in industry (French fry/crisp suitability and starch content) show independence regarding berry production, as observed for the varieties Sandra and Granola, for example. Knowledge on the varieties berry productivity would be important for the establishment of a side stream utilization of potato berries and possible future choice of cultivars producing berries.

### 2.3 Potato Metabolites

The potato metabolome includes diverse classes of primary and secondary metabolites with different activities, including fatty acids, alkanols, and sterols.^81^ This metabolic composition can be different according to the specific variety and tissue, being unequally distributed throughout the potato plant.^82^

Most research related to potato metabolites refers to tubers,^83−88^ whereas the aboveground tissues have received comparably little attention. Phenolic compounds such as chlorogenic acid, its isomer, and caffeic acid are present in higher amounts in flowers (in total 626 mg/100 g fw) than in leaves (in total 29.3 mg/100 g fw) and stems (in total 10.7 mg/100 g fw).^89^ Flavonoid contents like those of rutin also vary depending on the tissue analyzed, with ranges of 30 to 60 μg/g fw in leaves and 0.6 to 0.8 μg/g fw in stems.^90^ In tubers, phenolic compounds are more concentrated in the skin than in the flesh,^91^ but different species of potato with varied tuber coloration may also present differences in the amounts of phenolic compounds.^92^ According to Payyavula et al., anthocyanins, for example, are compounds easily found in tubers with purple coloration, ranging from 6.3 to 10 mg/g dw, depending on the tuber age (mature tubers containing lower amounts than immature tubers).^92^ Chlorogenic acid and flavonols can also be found in higher concentrations in purple-colored potatoes than in yellow and white ones.^92^

Potato secondary metabolites function variously, for example, as attractants, defense compounds, as antioxidants, and UV light protectants.^93,94^ Stress situations trigger the accumulation of secondary metabolites.^95−98^ According to Isah, factors such as presence of herbivores, lack of irrigation, light intensity, temperature variations, and salt concentration are responsible for activating defense responses.^97^ These are controlled by physiological changes that induce the production of secondary metabolites precursors, culminating later in accumulation of these molecules.^95−98^ Younger leaves and tissues of the native South American potato (*Solanum tuberosum* of the phureja group) contain high amounts of secondary metabolites related to plant defense, which increase the resistance of more vulnerable and highly metabolically active organs to attack of pests and pathogens.^99^

The nitrogen-containing metabolites in potato include alkaloids, one of the largest classes of secondary metabolites in higher plants, which have been used as traditional medicines since antiquity.^95^ The alkaloids can be divided into true alkaloids, pseudoalkaloids, and protoalkaloids, according to the precursor molecule and ring type (Table 2). More than 15 000 alkaloids have been identified in the families *Liliaceae, Solanaceae* and *Apocynaceae*.^94,100,101^

**Table 2:** Main Differences among Alkaloid Groups^a^

alkaloid class	heterocyclic ring	precursor	examples
true alkaloids	nitrogen-containing heterocyclic ring	l-phenylalanine, l-tyrosine, l-ornithine, l-histidine, l-lysine	nicotine, cocaine, morphine
pseudoalkaloids	no heterocyclic ring	nonamino acids	capsaicin, caffeine, ephedrine
protoalkaloids	no heterocyclic ring, nitrogen atom present	L-tryptophan, l-tyrosine	mescaline

a Source: Adaptation from Bennet and Wallsgrove^100^ and Dey et al.^101^

#### 2.3.1 Solanaceous Glycoalkaloids

**Figure 1:**
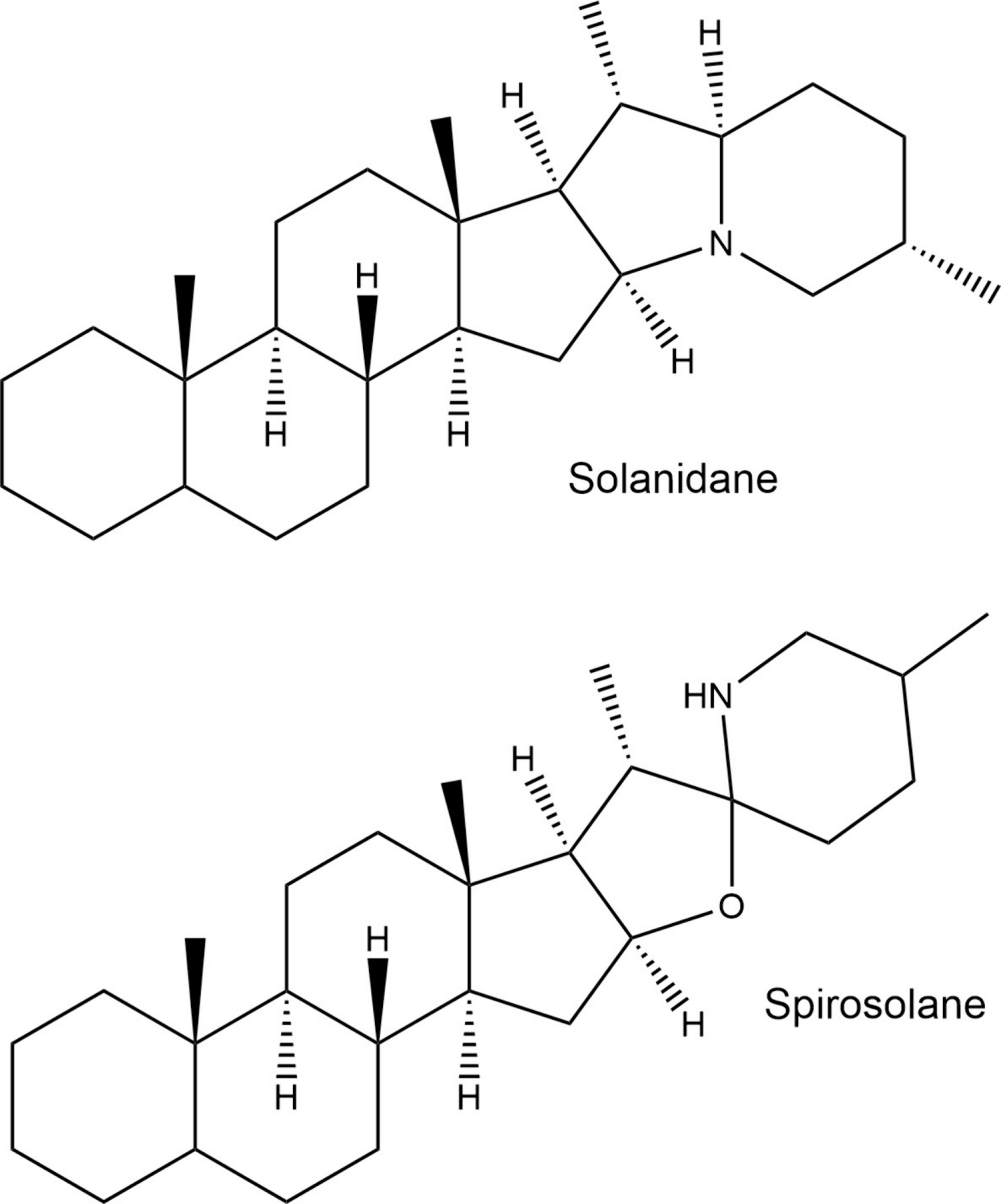
Chemical structure of major aglycones found in solanaceous plants.

Glycoalkaloids (steroidal glycoalkaloids) are pseudoalkaloids found mostly in solanaceous plants, including potato. These compounds are derived from the mevalonate and sesquiterpenoid pathways^102^ and comprise two main structural parts that confer amphiphilic characteristics.^103^ The hydrophobic portion is an aglycone, a C27 steroidal skeleton that includes a nitrogen atom in the ring derived from amino acids. The hydrophilic portion is a carbohydrate moiety attached to the 3-OH position of the aglycone.^103,104^ Depending on the arrangement of the rings (indolizidine or oxa-azaspirodecane ring system), different types of aglycones can be formed during the biosynthesis of glycoalkaloids. These differences produce two major forms of aglycones: solanidane and spirosolane (Figure 1). Examples of solanidane aglycones include solanidine and demissidine, whereas the spirosolane group includes tomatidine and solasodine.^105^ Carbohydrate side chains are present as 3–5 sugars attached to aglycones in different conformations.^106^ The most common monosaccharides are d-glucose, d-xylose, d-galactose, and l-rhamnose.^104^

The biosynthesis of glycoalkaloids has cholesterol as precursor. While in other plants the presence of cholesterol is not so striking, in solanaceous plants including potato this molecule can correspond to up to 20% of the 4-desmethyl sterols.^107^ Once formed from acetyl-CoA, cholesterol is then converted stepwise by hydroxylation, oxidation, and transamination into the aglycones, which are then glycosylated.^108−111^ This involves a series of enzymatic steps.^102,105,112,113^ Previous studies^113−115^ showed that knockdown of genes related to the biosynthesis of precursors and critical enzymes such as hydroxylation catalyzers (cytochrome P450 monooxygenases) reduce the levels of glycoalkaloids without affecting the vegetative growth and tuber yield.

After glycosylation, the intact glycoalkaloid is in the α-form and described as an α-glycoalkaloid. Enzymatic hydrolysis of the glycosidic part changes the molecule conformation resulting in shorter forms: β-, γ-, and δ-glycoalkaloids.^103,116^ In potato, up to 95% of the total glycoalkaloid content is represented by the α-conformation of solanine and chaconine which are formed from the aglycone solanidine.^105^ Carrying the same stereochemistry as its precursor cholesterol, solanidine is a solanidane type of aglycone that can be used to synthesize hormones^117^ and other biologically active compounds.^105^ Akiyama et al. reported that solanidane aglycones are mostly synthesized via the modification of spirosolane aglycones. According to these authors, differences in the activity of dioxygenase enzymes generate interspecies diversity, although the metabolic pathway is well conserved within the genus *Solanum*.^110^ In addition, Omayio et al. state that distinct glycoalkaloids are formed by different functional groups, C–C double bonds, and others. In potato, this diversity is generated by the carbohydrate moieties.^118^

**Figure 2:**
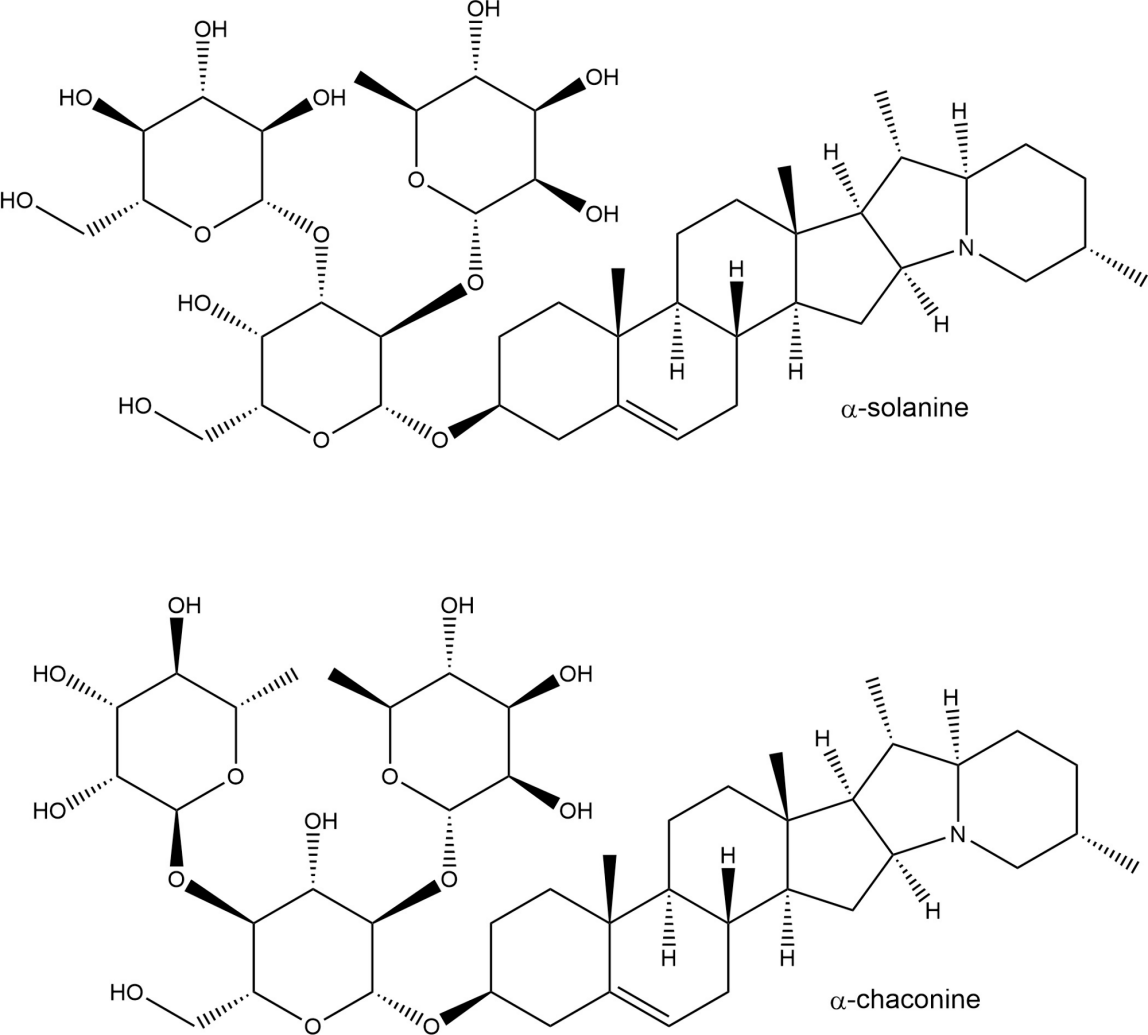
Chemical structure of potato glycoalkaloids.

Solanidine can be glycosylated at the 3-OH position by two main carbohydrate structures. The first is the trisaccharide β-solatriose, comprising galactose, rhamnose, and glucose units, producing the glycoalkaloid α-solanine (also known as (3β)-solanid-5-en-3-yl 6-deoxy-α-l-mannopyranosyl-(1→2)-[β-d-glucopyranosyl-(1→3)]-β-d-galactopyranoside; C_45_H_73_NO_15_). The second is the trisaccharide β chacotriose, comprising one glucose and two rhamnose units, producing the glycoalkaloid α-chaconine (also known as (3β)-solanid-5-en-3-yl-6-deoxy-α-l-mannopyranosyl-(1→2)-[6-deoxy-α-l-mannopyranosyl-(1→4)]-β-d-glucopyranoside; C_45_H_73_NO_14_)^119^ (Figure 2). Potato tissues also contain low levels (∼5%) of hydrolysis products such as β and γ solanine/chaconine, as well as the aglycone solanidine itself.^120^

Even with described antifungal,^121^ antiviral,^122^ and antitumor^123^ activities for the major potato glycoalkaloids α-solanine and α-chaconine, the presence of distinct carbohydrate side chains can affect their performance. Some authors report that glycoalkaloids with chacotriose side chains are more active than those carrying solatriose,^124^ suggesting α-chaconine as the more active compound. This was manifested, e.g., in the higher cytotoxicity of α-chaconine *in vitro*, which may reflect the ability of rhamnose to promote uptake by cell-surface receptors.^125^

A possible explanation for the existence of two different glycoalkaloids differing in activity is the coevolution of potato with pests and pathogens. As mentioned before, alkaloids can be often related to defense mechanisms. Therefore, Friedman suggested that the emergence of a second glycoalkaloid (α-chaconine), in addition to α-solanine, would have followed the adaptation of biotic antagonists to α-solanine, and the necessity to overcome this mechanism of resistance.^120^

Glycoalkaloids are formed during the entire developmental cycle of potato plants.^120^ Also, glycoalkaloid accumulation is influenced by a combination of genetic and environmental factors acting at the level of the whole plant or individual organs.^126−128^ However, the environmental impact on accumulation of glycoalkaloids in potato tubers has received the most attention in this regard. Tajner-Czopek et al. listed a series of abiotic factors that can increase the level of glycoalkaloids in potato tubers, such as access to light, mechanical damage, and poor storage conditions. Furthermore, doubling the rate of nitrogen fertilizer application increased the TGA by up to 10%.^129^ Machado et al. studied the effect of light intensity on glycoalkaloid accumulation but did not find any differences between indirect sunlight and fluorescent light for the cultivar Monaliza, and indeed observed the accumulation of glycoalkaloids regardless of the presence of light.^130^ Storage temperature might affect the accumulation of glycoalkaloids in potato tubers. Griffiths et al. showed that tubers stored at 4 °C accumulate larger amount of TGA than tubers stored at 10 °C for the varieties Brodick and Pentland Crown. This concentration also increases according to the time the tubers are kept at this temperature.^131^ Later, the authors observed that storage in cold temperatures followed by light exposure also contributes to the increase in the content of glycoalkaloids.^132^ Drought is also described as an abiotic factor responsible for the accumulation of glycoalkaloids in potato. According to Bejarano et al., the lack of water can increase in up to 75% the TGA of tubers from the variety Desiree, without exceeding the recommended maximum food safety concentration of 200 mg/kg fw.^133^ Interestingly, fungal infection can also act as trigger for the biosynthesis of alkaloids. Aliferis and Jabaji observed that sprouts infected with *Rhizoctonia solani* had increased quantities of solasonine and solasodine.^134^

The typical α-chaconine:α-solanine ratio is 60:40,^135^ but this differs between potato varieties.^136^ Although α-chaconine has a greater impact on total glycoalkaloid levels in potato tissues, it is broken down more rapidly than α-solanine.^126^ Glycoalkaloids are found in potato leaves, roots, tubers, peel, flowers, sprouts, and fruits, but the highest levels are found in particularly vulnerable tissues with high metabolic activity, such as flowers and sprouts, reflecting the need for an effective defense mechanism against pests and diseases.^119,126,127,135^ An average of the α-solanine and α-chaconine content in different potato tissues is summarized in Table 3.^137^

**Table 3:** Glycoalkaloid Content in Different Potato Tissues Described by Friedman and Dao^a^,^137^

potato tissue	α-chaconine (mg/100 g fw)	α-solanine (mg/100 g fw)
tubers	9.27	5.35
berries	22.1	15.9
roots	44.57	41.19
leaves	84.57	60.51
sprouts	520.18	476.88

a Source: Adapted from Friedman and Dao.^137^

**Figure 3:**
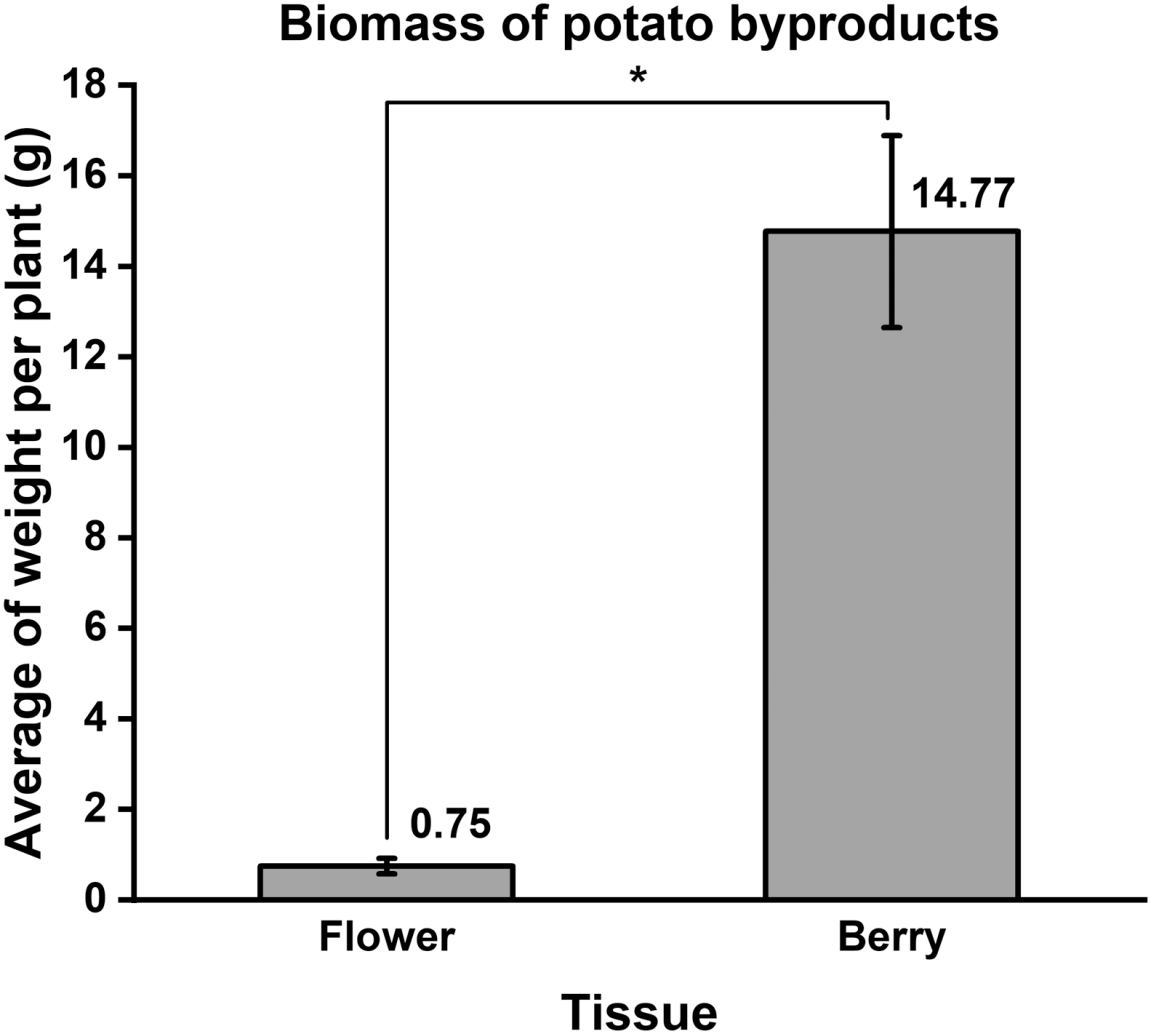
Fresh weight of flowers and berries harvested in a field trial from the potato variety Quarta. Error bars indicate SE for *n* = 4 blocks, and asterisk (*) shows significant difference between the tissues by *t* test (at 5% of significance). Source: The Authors.

Potato berries contain lower levels of glycoalkaloids than flowers, but 10–20 times more than tubers.^127,138^ The biomass of flowers is much lower than for berries (Figure 3), therefore berries could be well used as a source for glycoalkaloids.

Glycoalkaloid levels vary significantly among berries from different potato cultivars, ranging from 17.7 mg TGA/100 g fw in Maris Peer to 135.4 mg TGA/100 g fw on Record.^138^

In general, leaves have a high content of glycoalkaloids, however, potato plants are usually grown until senescence before harvest of tubers^139^ and, with senescence, the content of glycoalkaloids is strongly reduced.^126^ Therefore, the content decreases as the plants develop and the berries increase in size.^127,135^

Tubers of commercial cultivars contain less than 100 mg of glycoalkaloids/kg fw.^140^ Potato tubers should not be consumed when glycoalkaloid levels exceed 200 mg/kg fw.^141^ Higher concentrations (above 140 mg/kg fw) give the tubers a bitter taste and induce gastroenterological and neurotoxic reactions,^142,143^ such as nausea, vomiting, diarrhea, abdominal cramps, hallucinations, and in more severe cases coma.^144^ As the only edible part of a potato plant, the glycoalkaloid content of cultivated tubers must be kept as low as possible, particularly because these compounds are not destroyed by cooking.^133,145^ Breeding programs have therefore focused on reducing the level of glycoalkaloids in tubers while maintaining biotic and abiotic stress resistance traits.^146,147^ However, the parental lines must be selected carefully because glycoalkaloid production is a heritable characteristic.^120,148^*Solanum chacoense* is one example of a wild tuber-bearing *Solanum* species used in breeding programs. This wild potato is a promising candidate for breeding more tolerant potato cultivars, because of its extreme tolerance against adverse conditions due to the high content of leptins, acetylated forms of chaconine, and solanine.^149,150^ For commercialized potato products, Friedman and Dao observed that French fries contained a smaller amount of total glycoalkaloids (0.84 mg of TGA/100 g of product) when compared to potato chips (up to 10.90 mg of TGA/100 g of product).^137^ Bushway and Ponnampalam explain that the glycoalkaloid content of potato products, e.g., potato chips and frozen French fries, may vary according to the manufacturing process, where storage conditions and processing might influence the final content.^145^

Although the negative effects of glycoalkaloid consumption are well-known,^128,151−153^ they also have many beneficial properties. Therefore, this class of secondary metabolites should not be solely regarded as a problem but also as a potential solution, as discussed in the next section.

## 3 Berries of the *Solanum* Genus and Their Different Bioactivities

**Figure 4:**
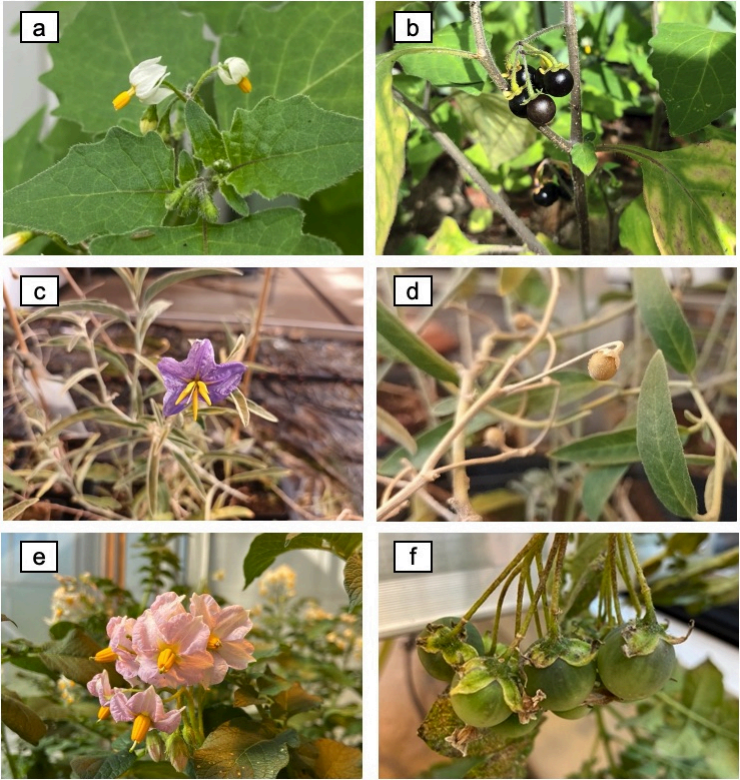
Morphological similarities of flower and berry from three species of *Solanum* genus: (a,b) *Solanum nigrum*, (c,d) , (e,f) *Solanum tuberosum*. (a–f) Authors’ directory.

*Solanum* is the largest genus of solanaceous plants, comprising ∼1200 species,^154^ which have been intensively explored to produce food and pharmaceuticals due to the presence of highly valuable active compounds.^155^Figure 4 shows similarities between solanaceous species in terms of flower and berry morphologies. This section shows the bioactivity of berries from other solanaceous species as a way of suggesting an application of potato berries for the same purposes. Thus, it may be assumed that compounds present in potato berries have similar bioactivity like the compounds present in other nightshades. In addition, a few examples of similar compounds present in other potato tissues and berries were also included to emphasize this link.

In terms of pharmaceutical purposes, anti-inflammatory, anticarcinogenic, and antioxidant activities of alkaloids from *Solanum* species have been previously reported and are described next. Previous research on *Solanum* extracts found compounds that inhibit the development of carcinogenic cells. For example, *Solanum aculeastrum* is a plant used in South Africa for the treatment of breast cancer. Koduru et al. reported the inhibition of cancer cell growth by more than 80% in 48 h following the application of *S. aculeastrum* extracts *in vitro*. The positive results were related to berry extracts, while leaf extracts did not promote antitumor activity.^156^ Later, the authors found that the presence of alkaloids (tomatidine and solasodine) in berry tissue, and the positive synergy between them enhanced their inhibitory activity on cell growth.^157^

The antioxidant activity of metabolites is often exploited in product research and development. For example, berry extracts of *Solanum aculeastrum*(158) and *Solanum nigrum*(159) contain high levels of polyphenols, which can scavenge and inactivate reactive oxygen species.^160^*S. nigrum* (Figure 4) is extensively used in traditional medicine to treat gastrointestinal illnesses, heart conditions, and skin wounds.^161^

Anti-inflammatory drugs are also one of the main targets of the pharmaceutical industry. Extracts of *Solanum nigrum* (100 and 200 mg/kg) were shown to reduce edema in mice by 38.4% and 44.8%, respectively,^161^ which could be caused by flavonoids^161^ or which may reflect the anti-inflammatory effect of steroidal alkaloids in berries of *S. nigrum*.^162,163^ Anosike et al. supplemented mouse diets with *Solanum aethiopicum* berries at different concentrations (5, 10, and 20%). After 5 h, mice on the 20% berry diet composed showed an 81.8% reduction in edema.^164^

Endocrine and respiratory diseases could be alleviated by the administration of *Solanum* extracts. For example, extracts of *S. nigrum* berries^165^ reduced glucose levels in the blood of diabetic patients. Again, this may reflect the presence of alkaloids, which can stimulate the release of insulin in the pancreas.^166^ Above-ground extracts (leaves, stems, flowers, and fruits) of *Solanum xanthocarpum* and *Solanum trilobatum* induce positive bronchodilator action, thus reducing the impact of bronchial asthma.^167^

*Solanum* alkaloids in general have also shown potential for utilization in control of various mammalian pathogens. Herpes simplex virus Type 1, for example, was the target for different studies in the past. Thorne et al. observed that glycoalkaloids with different sugar moieties act distinctly in inactivating the virus in tissue culture. While α-chaconine with two rhamnose units was already effective at 0.01%, α-solasonine (one rhamnose and one glucose units) and α-tomatine (two glucose and one xylose units) were effective only a 10-fold higher concentration, while α-solanine (one rhamnose and one glucose units) was not effective.^122^ According to the authors, the sugar moiety plays an important role in the inactivation with its interaction with the viral envelope.

Ikeda et al. performed a study with testing the action of a few glycoalkaloids extracted from different *Solanum* species and tissues against herpes simplex virus 1.^168^ Their results showed that other steroidal alkaloids obtained from immature fruits of *S. nigrum* and a nuatigenin type glycoside from fruits of *S. abutiloides* were also compounds effective against herpes simplex virus type 1 (EC_50_ of 1.95 and 2.70 μg/mL, respectively).^168^

Among bacteria, Gram-positive species appear more susceptible to the metabolites in *Solanum* plants than Gram-negative species. Amanpour et al. inhibited the growth of *Staphylococcus aureus* (Gram-positive) by applying peel extracts at a concentration of 0.62 mg/mL, whereas the growth of *Klebsiella pneumoniae* (Gram-negative) was unaffected, even at a concentration of 10 mg/mL.^169^ Bacterial inhibition is also influenced by the tissue used to prepare extracts, probably reflecting the different metabolic compositions. Sridhar et al. found that *Solanum nigrum* seed extracts performed better than leaf and root extracts.^170^

*Solanum* extracts often have a dose-dependent effect on pathogenicity, with growth impairment directly proportional to the extract concentration.^171^*Candida albicans* is influenced by alkaloids from *Solanum congestiflorum* berries. Kusano et al. found that compounds such as solacongestidine, verazine, and solafloridine inhibit the synthesis of molecules required for fungal growth, such as cholesterol and ergosterol. *Solanum torvum* and *Solanum incanum* berry extracts also inhibited the growth of *Trichophyton rubrum* and *C. albicans*.^172^

*Solanum* extracts also show molluscicide activity, making them useful for the control of parasitic diseases transmitted by snails.^173^ For example, *Solanum aculeastrum* berry extracts tested against *Biomphalaria pfeifferi* (the host of the parasite *Schistosoma mansoni*, which causes schistosomiasis) induced 100% mortality at concentrations of 50 ppm and 85% mortality at 10 ppm.^174^*S. aculeastrum* berry extracts contain the alkaloids β-solamarine and solamargine, which act synergistically and therefore induce 100% mortality at concentrations as low as 8 ppm.^175^ Silva et al. showed that pure solamargine achieved an LC_50_ value of 26.3 μg/mL, whereas crude extracts containing this alkaloid were almost three times more potent, with an LC_50_ value of 9.7 μg/mL.^176^ Mixtures of the glycoalkaloids solamargine and solasonine from *Solanum lycocarpum* berry extracts also induced 100% mortality in the parasite itself, *Schistosoma mansoni*.^177^

The mosquitoes *Aedes aegypti* and *Stegomyia aegypti*, which transmit dengue fever to humans, are also susceptible to *Solanum* extracts. Different concentrations of *Solanum villosum* berry extract (aqueous) was also tested against populations of *S. aegyptii*. For a period of 72 h, the highest mortality was induced by the highest concentration (0.5%), while the lowest concentration (0.1%) induced a mortality of only 30%.^178^

Besides the potential for utilization in development of pharmaceuticals, *Solanum* species contain metabolites showing cytotoxic and deterrent properties, making them interesting for an application in development of plant protection products. For instance, the glycoalkaloid α-chaconine from potato is active against nematodes, although a higher dosage is required in acidic soils.^179^ Khan et al. showed that chloroform extracts of *Solanum nigrum* leaves also have nematicidal activity, with an LD_50_ value of 1.21 mg/mL against *Caenorhabditis elegans*.^180^ The same extracts also immobilize and kill *Pratylenchus goodeyi*.^181^ Moreover, bioactivity of various alkaloids of *Solanum* species has been observed against several pests. Feeding deterrent experiments with *Solanum eleagnifolium* seed extracts containing the alkaloids solamargine, solasonine, and solasodine for example have been conducted with the red flour beetle *Tribolium castaneum*.^182^ The extracts induced 88% mortality following ingestion after treatment for 7 days and achieved 94% repellent efficacy after 2 h.^183^ Another alkaloid (luciamin) from *Solanum laxum* was shown to deter the aphid *Schizaphis graminum*, a common pest in grasses.^184^

Zouiten et al. reported the insecticidal activity of *Solanum sodomaeum* berry skin extracts against the desert locust *Schistocerca gregaria*, with 50% of larvae killed after 5 days, due mainly to developmental and neurological defects.^185^*Solanum mammosum* berry extracts induced 59.5% mortality in the fruit fly *Drosophila melanogaster*, with an LC_50_ value of 80 mg/mL, and disrupted the formation of pupation in 48.3% of the affected individuals.^186^

The effect of bioactive compounds can be increased by more effective delivery mechanisms, such as the use of nanoparticles. Almadiy and Nenaah showed that potato leaf extracts combined with nanoparticles enhanced the antifungal effect against *Alternaria alternate, Rhizoctonia solani, Botrytis cinerea*, and *Fusarium oxysporum* f.sp. *lycopersici*.^187^ They tested α-solanine and α-chaconine alone, in a mixture, or in a formulation with silver nanoparticles, and found that *R. solani* mycelial growth was inhibited more by the mixture and the formulation, suggesting the synergistic activity of the components.^187^

In conclusion, the presence of compounds in berries from various solanaceous plants, which are highly valuable for utilization in pharmaceutical industry or plant protection products, suggests the possibility of the same value and usability for berries of *S. tuberosum*. While other *Solanum* species are widely used in traditional medicine due to their anticancer, antimicrobial, and anti-inflammatory activities, potato berries with glycoalkaloids showing similar activities are not yet investigated for such uses. This is due to the fact that besides a few studies on glycoalkaloid content,^137^ there is not much knowledge on the metabolic composition of potato berries. This review suggests that more attention should be paid to potato berries so that glycoalkaloids and other secondary metabolites present in this free and unused field residue are better exploited. Therefore, farmers can take advantage of another source of income within the same agricultural crop land they use to produce their tubers. Furthermore, future breeding of potato might aim an effective production of berries as a further trait.

## Abbreviations

C–C: carbon–carbon bond
EC_50_: half-maximal effective concentration
ECPD: European Cultivated Potato Database
EU: European Union
kJ: kilojoule
LC_50_: concentration required to kill 50% of the tested sample
SNP: single nucleotide polymorphism
SS: suspended solid
TGA: total glycoalkaloid content
UN: United Nations
USA: United States of America
